# Intratonsillar Immunotherapy: A Convenient and Effective Alternative to Subcutaneous Immunotherapy for Allergic Rhinitis

**DOI:** 10.34133/research.0573

**Published:** 2025-01-16

**Authors:** Tian Gu, Wei Zhang, Lu Tan, Rong Xiang, Peiqiang Liu, Jingyu Huang, Qin Deng, Yuqin Deng, Zezhang Tao, Shiming Chen, Yu Xu

**Affiliations:** ^1^ Department of Rhinology and Allergy, Otolaryngology-Head and Neck Surgery Center, Renmin Hospital of Wuhan University, Wuhan 430060, China.; ^2^ Research Institute of Otolaryngology-Head and Neck Surgery, Renmin Hospital of Wuhan University, Wuhan, China.; ^3^ Hubei Province Key Laboratory of Allergy and Immunity, Wuhan, China.

## Abstract

Allergen-specific immunotherapy (AIT) is the only treatment that addresses the root cause of immunoglobulin E (IgE)-mediated allergies, but conventional methods face challenges with treatment duration, patient compliance, and adverse effects. In this study, we propose intratonsillar immunotherapy (ITIT) as a new effective and safer route for AIT. Prior to clinical trials, we analyzed tonsil samples from human subjects to assess immune responses, measuring interleukin-4 (IL-4), IL-21, total IgE (tIgE), and allergen-specific IgE concentrations using ELISA and BioIC. Our results indicated that tonsils contained higher levels of allergen-specific IgE compared to peripheral blood. In the clinical phase, 120 allergic rhinitis (AR) patients were treated with either 3 intratonsillar allergen injections over 2 months or conventional subcutaneous immunotherapy (SCIT) over 1 year. ITIT demonstrated superior and faster symptom relief, especially in younger patients, while requiring markedly fewer doses and injections than SCIT. Immunological analysis revealed reduced eosinophil counts, increased regulatory T (T_reg_) and follicular regulatory T (T_FR_) cell levels, and a favorable shift in cytokine profiles. Adverse events were minimal, and the treatment showed high patient compliance. These findings suggest that ITIT could provide an effective, safer, and more convenient alternative to AIT.

## Introduction

Allergic diseases, which importantly affect the quality of life and result in a considerable economic burden, are a growing global health concern [1]. Allergen-specific immunotherapy (AIT) remains the only cause treatment, offering sustained symptom relief and preventing disease progression. However, challenges such as lengthy treatment duration, side effects, and low patient compliance limit its broader adoption [2]. In response, researchers have explored alternative methods for delivering AIT to improve effectiveness and patient convenience [3]. Among these, intralymphatic immunotherapy (ILIT) has shown promise by administering allergens directly into the lymph nodes, thereby inducing rapid immune responses with smaller allergen doses [4]. Studies have confirmed its effectiveness [5–10]. However, ILIT is challenging because lymph nodes are not easily accessible and require ultrasound-guided injections, which need high surgical skill and carry the risk of serious side effects [11,12]. These issues emphasize the need to continue exploring and developing reliable treatment methods.

The tonsils, located near the nasal cavity, function as secondary lymphoid organs, mediating immune responses to allergens in the nasal region [13]. Unlike lymph nodes, tonsils lack encapsulation and afferent lymphatics, but their reticular crypt epithelium contains dendritic cells that transport antigens to T and B cell zones, initiating immune responses [14]. Due to their proximity to the respiratory tract, allergen injections into the tonsils can elicit rapid and localized immune responses, similar to ILIT but with fewer associated risks [15]. Additionally, the mucosal surface of the tonsils contains very few mast cells, significantly reducing the risk of local allergic reactions [16,17]. The direct accessibility of the tonsils via the oral cavity allows for convenient and precise therapeutic injections. This ease of access makes intratonsillar immunotherapy (ITIT) a practical and efficient route for AIT, offering potential advantages in both safety and administration.

In this study, we propose a novel approach: administering allergen extracts through intratonsillar injections over a 2-month period. We compare the efficacy and safety of ITIT with the established subcutaneous immunotherapy (SCIT) over 1 year, and explore immune responses and influencing factors associated with this new treatment.

## Results

### Tonsil involvement in the immune response of allergic rhinitis

Allergy is recognized as a risk factor for tonsil hypertrophy [18]. Recent studies highlight significant differences in tonsil transcriptomes and microbiomes between allergic and nonallergic individuals, suggesting that the tonsils are involved in sensitization as secondary lymphoid tissue [19,20]. To investigate this further, we measured the levels of allergy-related substances in tonsil tissues from 46 participants (median age, 9 years; range, 5 to 51 years) without intervention. Participants were divided into adult (*n* = 13) and pediatric (*n* = 31) groups to explore age-related differences in the immune response. Allergic rhinitis (AR) was confirmed through skin prick test (SPT) and/or serum immunoglobulin E (sIgE) levels, along with nasal symptoms. Of the 37 participants, 24 (52.2%) had dust mite-induced AR, and 22 (47.8%) were non-atopic.

**Fig. 1.**
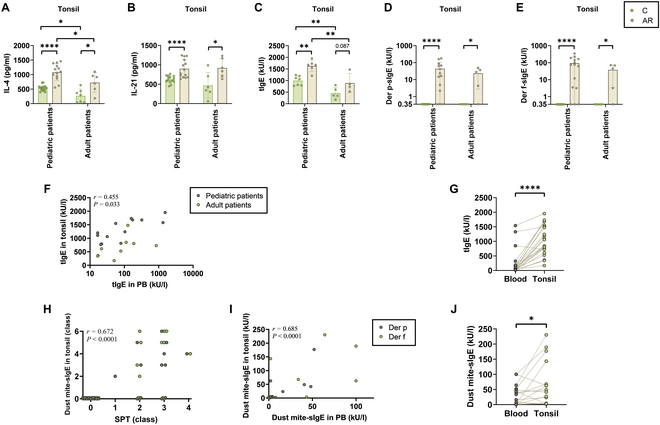
Expression of immune markers in tonsils of AR patients and non-atopic individuals. (A) IL-4, (B) IL-21, (C) tIgE, (D) Der p-sIgE, and (E) Der f-sIgE in tonsil of AR patients and control individuals are demonstrated. (F) Correlation of tIgE in tonsil and peripheral blood. (G) Comparison of tIgE in tonsil and peripheral blood. (H) Correlation of sIgE in tonsil with class of SPT. (I) Correlation of sIgE in tonsil and peripheral blood. (J) Comparison of sIgE in tonsil and peripheral blood. Some patients were evaluated for dust mite-induced AR using either the SPT or serum sIgE test, but not both. ****P < 0.0001, **P < 0.01, and *P < 0.05. C, control group; AR, allergic rhinitis; tIgE, total IgE; sIgE, specific IgE; SPT, skin prick test; PB, peripheral blood.

In both adult and pediatric groups, allergic patients exhibited higher levels of the type 2 cytokine interleukin-4 (IL-4), the follicular helper T (T_FH_) cell cytokine IL-21, tIgE, and dust mite-sIgE in their tonsils compared to nonallergic individuals. Additionally, younger individuals exhibited higher levels of IL-4 and tIgE in their tonsils (Fig. 1A to E). These findings suggest that while tonsillar tissue undergoes age-related degeneration, adult tonsils still retain considerable immunological activity in response to allergens and AR.

Furthermore, tonsillar tIgE levels were significantly higher than those in peripheral blood and showed a positive correlation (Fig. 1F and G). Similarly, tonsillar dust mite-sIgE levels were elevated relative to peripheral blood sIgE levels and showed a positive correlation with both SPT and peripheral sIgE (Fig. 1H to J). These findings support the close association of tonsils with the presence and severity of nasal allergy.

Taken together, the elevated IgE levels and cytokine responses in the tonsils, alongside their correlation with systemic immune markers, highlight the tonsils as a critical site for initiating and modulating immune responses in AR. This suggests that tonsillar injection may provide a promising therapeutic avenue for improving AR treatment outcomes.

### Clinical demographics

A total of 120 dust mite allergy-induced AR patients were enrolled, with 60 patients in each treatment group (ITIT or SCIT), based on their preference. Enrollment for each group was closed once it reached full capacity. Baseline characteristics—gender, age, medical history, symptom scores (SSs), and medication scores (MSs)—were comparable between the 2 groups (Table 1). We performed a secondary analysis after adjusting for sIgE levels, and the results showed that sIgE did not affect the outcome of this cohort of patients (Table S1).

**Table 1.** The characteristics and laboratory findings at baseline of the SCIT and ITIT patients

Characteristic	SCIT	ITIT	Total	*P* value
Number of patients		60	60	120	
Female sex, *n* (%)		23 (38.33)	24 (40.00)	47 (39.17)	0.852
Age (years), median (IQR)		19.00 (10.00–31.75)	15.00 (11.00–24.75)	17.00 (8.00–33.50)	0.488
Age group, *n* (%)	≤18	29 (48.33)	37 (61.67)	66 (55.00)	0.142
CSMS, mean (SD)		1.86 (1.07)	1.68 (0.67)	1.73 (0.92)	0.852
SS, mean (SD)		1.13 (0.67)	1.26 (0.54)	1.19 (0.61)	0.269
MS, median (IQR)		0.29 (0.00–1.07)	0.20 (0.00–0.93)	0.25 (0.00–0.98)	0.323
VAS, mean (SD)		20.18 (11.68)	22.36 (9.74)	21.18 (11.07)	0.218
Family history of atopy, *n* (%)		12 (20.00)	17 (28.33)	29 (24.17)	0.286
History of drug allergy, *n* (%)		13 (21.67)	9 (15.00)	22 (18.33)	0.345
Monosensitization to dust mites, *n* (%)		41 (68.33)	38 (63.33)	79 (65.83)	0.564
Polysensitization to other allergens, *n* (%)	Animal dander	11 (18.33)	5 (8.33)	16 (26.67)	0.168
	Pollen	4 (6.67)	9 (15.00)	13 (21.67)	0.142
	Mold	1 (1.67)	3 (5.00)	4 (6.67)	0.309
	Cockroach	2 (3.33)	3 (5.00)	5 (8.33)	0.648
	Food	7 (11.67)	9 (15.00)	16 (26.67)	0.591
tIgE (pg/ml), median (IQR)		316.00 (109.00–706.00)	387.50 (180.25–849.00)	367.00 (169.00–791.50)	0.667
Der p-sIgE (kU/l), median (IQR)		19.86 (4.22–47.96)	45.14 (8.31–100.00)	27.51 (7.24–100.00)	0.019
Der f-sIgE (kU/l), median (IQR)		20.67 (6.17–73.89)	100.00 (17.08–100.00)	54.05 (10.15–100.00)	0.001
Dust mite-sIgE (kU/l), median (IQR)		34.91 (13.13–109.38)	141.47 (26.80–200.00)	99.24 (18.52–200.00)	0.003
Der p-sIgE/tIgE, median (IQR)		0.05 (0.02–0.14)	0.10 (0.03–0.17)	0.07 (0.03–0.14)	0.267
Der f-sIgE/tIgE, median (IQR)		0.08 (0.03–0.14)	0.12 (0.04–0.28)	0.12 (0.04–0.23)	0.053
Dust mite- sIgE/tIgE, median (IQR)		0.16 (0.06–0.30)	0.23 (0.06–0.53)	0.20 (0.06–0.35)	0.098
EOS, median (IQR)		0.29 (0.21–0.50)	0.36 (0.21–0.55)	0.33 (0.21–0.51)	0.443
EOS%, median (IQR)		4.45 (2.73–8.28)	5.40 (2.80–8.30)	5.20 (2.80–8.30)	0.431
BAS, median (IQR)		0.02 (0.02–0.05)	0.04 (0.02–0.06)	0.03 (0.02–0.05)	0.039
BAS%, median (IQR)		0.30 (0.20–0.70)	0.45 (0.30–0.80)	0.40 (0.30–0.80)	0.035
IgG, mean (SD)		11.24 (1.96)	11.85 (2.55)	11.59 (2.38)	0.303
IgA, mean (SD)		2.38 (0.95)	2.03 (0.85)	2.15 (0.91)	0.066

**Fig. 2.**
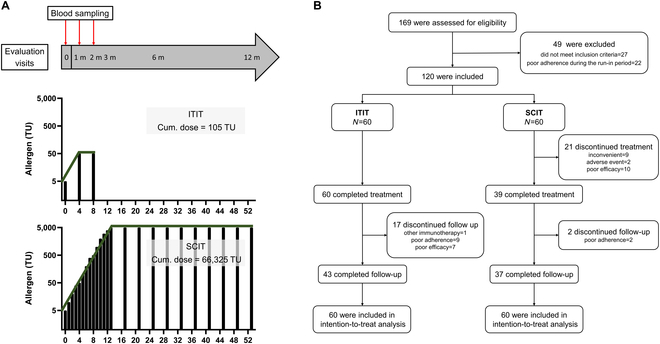
Clinical trial design. (A) Patients receiving ITIT undergo 3 injections within 8 weeks, with a cumulative dose of 105 TU. Patients receiving SCIT receive approximately 24 injections over the course of 1 year, with a cumulative allergen dose of 66,325 TU. Adverse reactions are assessed at each injection for all patients. Nasal questionnaire scores are evaluated at baseline, 1 month, 2 months, 3 months, 6 months, and 12 months, and immunological parameters are measured in the early stages of treatment (baseline, 1 month, 2 months). (B) Flow of patients. ITIT, intratonsillar immunotherapy; SCIT, subcutaneous immunotherapy.

Patients in both groups were treated and followed up according to the procedure shown in Fig. 2A. All ITIT patients completed their scheduled injections, but 17 were lost to follow-up by the end of the year. In the SCIT group, 21 patients did not complete the treatment, and 23 were lost to follow-up (Fig. 2B).

### Comparison of clinical efficacy between ITIT and SCIT

#### Enhanced efficacy of ITIT in early stages

**Fig. 3.**
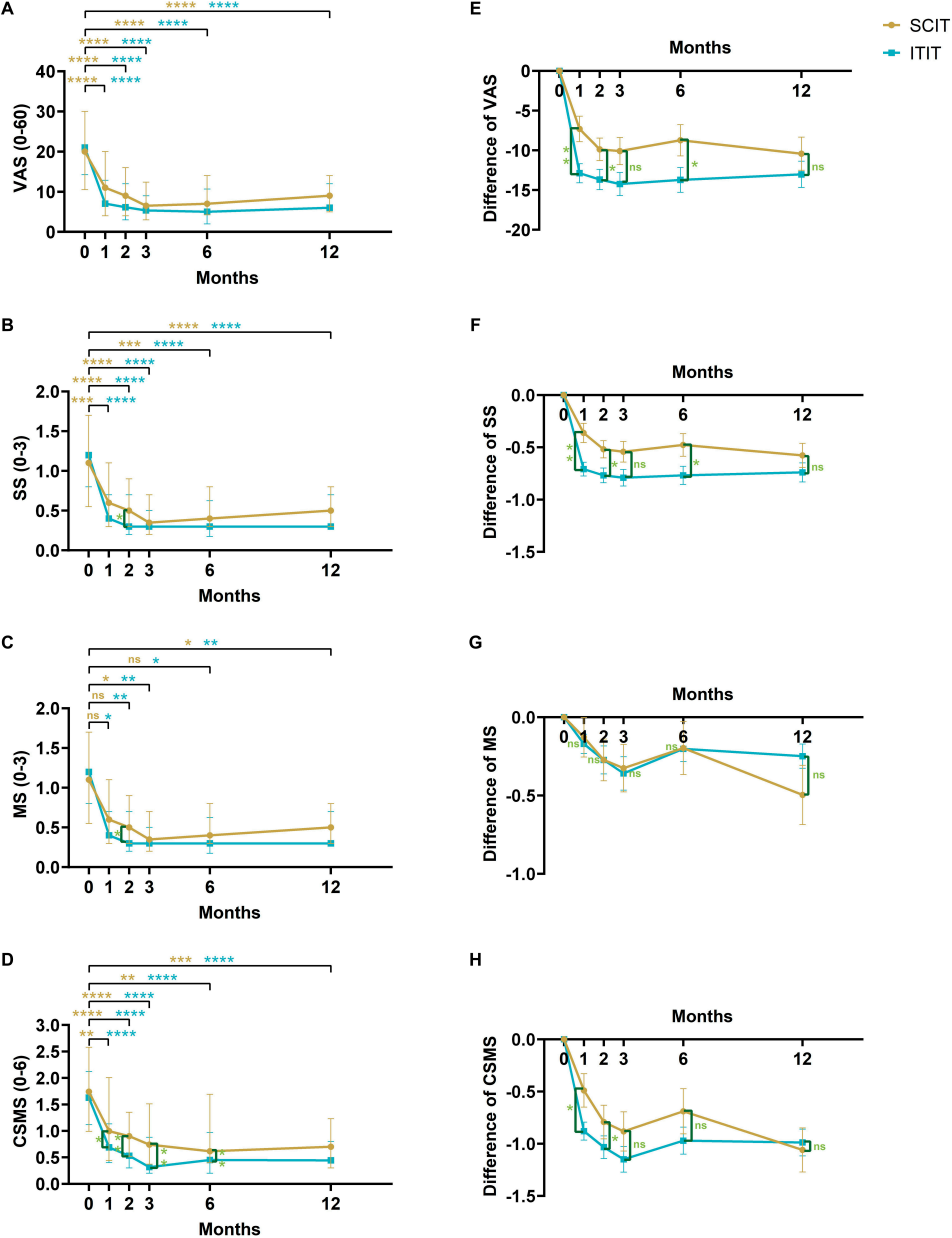
Comparison of overall efficacy between ITIT and SCIT. Changes in (A) VAS, (B) SS, (C) MS, and (D) CSMS of patients receiving ITIT and SCIT from baseline to 1, 2, 3, 6, and 12 months after treatment. Changes in (E) VAS, (F) SS, (G) MS, and (H) CSMS differences between the scores after treatment at each time interval and their corresponding baseline scores. ****P < 0.0001, ***P < 0.001, **P < 0.01, and *P < 0.05. ns represents *P* > 0.05. The specific *P* values are shown in Table S1. VAS, visual analog scale; SS, symptom score; MS, medication score; CSMS, combined symptom and medication score.

Visual analog scale (VAS), SS, MS, and combined symptom and medication score (CSMS) were used to assess treatment effectiveness. Both groups showed significant improvement in these measures across the 12-month period, although ITIT exhibited significantly better results in the early stages. At the 1-month mark, CSMS scores in the ITIT group were significantly lower than those in the SCIT group, and this advantage persisted until month 6. Both SS and MS scores were also significantly better in ITIT during the second month (Fig. 3A to D). The improvement in symptoms and combined scores in the ITIT group was significantly superior to that in the SCIT group at 1 month of treatment, and this superiority was sustained up to 6 months, too. Both groups exhibited similar improvements at the 12-month mark, and the improvement in MS scores was comparable during the follow-up period (Fig. 3E to H). The model adjusted for the covariate still supports these conclusions (Table S1).

#### Sustained efficacy in more patients of ITIT after 1 year

**Fig. 4.**
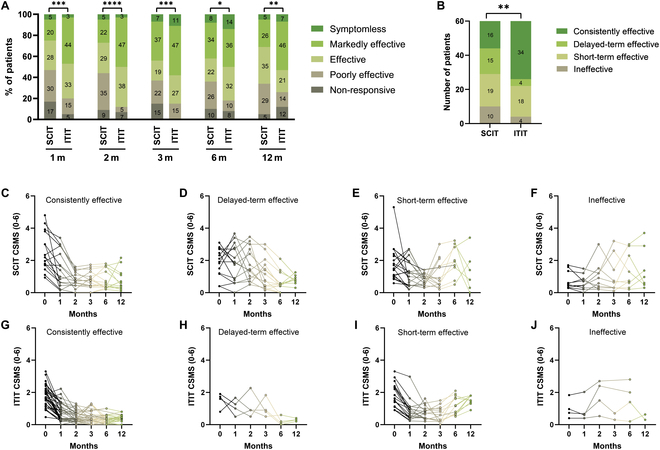
Stratification and dynamic analysis of CSMS changes after treatment. (A) Changes in the CSMS at each stage were divided into 5 groups based on the TI of each patient at different follow-up points. (B) Overview of patient classification under both treatment modalities. Patients were grouped into 4 categories based on the duration of their CSMS improvement: consistently effective, delayed-term effective, short-term effective, and ineffective (C to J). ****P < 0.0001, ***P < 0.001, **P < 0.01, and *P < 0.05.

To delve deeper into the differences in efficacy between SCIT and ITIT, we compared the response rates of the 2 treatments at each follow-up time point using the therapeutic index (TI) (refer to the “Primary endpoint” section for detailed methods). It showed a higher proportion of sustained efficacy in the ITIT group at 1 year compared to the SCIT group. At each time point, the distribution of the efficacy constituent ratios differed significantly between the 2 groups (Fig. 4A).

We then categorized patients into 4 subgroups to analyze the dynamic characteristics of symptom improvement (also detailed in the “Primary endpoint” section), providing a comprehensive view of the CSMS changes in each patient (Fig. 4B). The constituent ratio distribution of subgroups showed significant differences between the 2 treatment groups. ITIT consistently showed better outcomes, with more patients demonstrating sustained symptom relief (Fig. 4C to J).

#### Younger patients respond better to ITIT

**Fig. 5.**
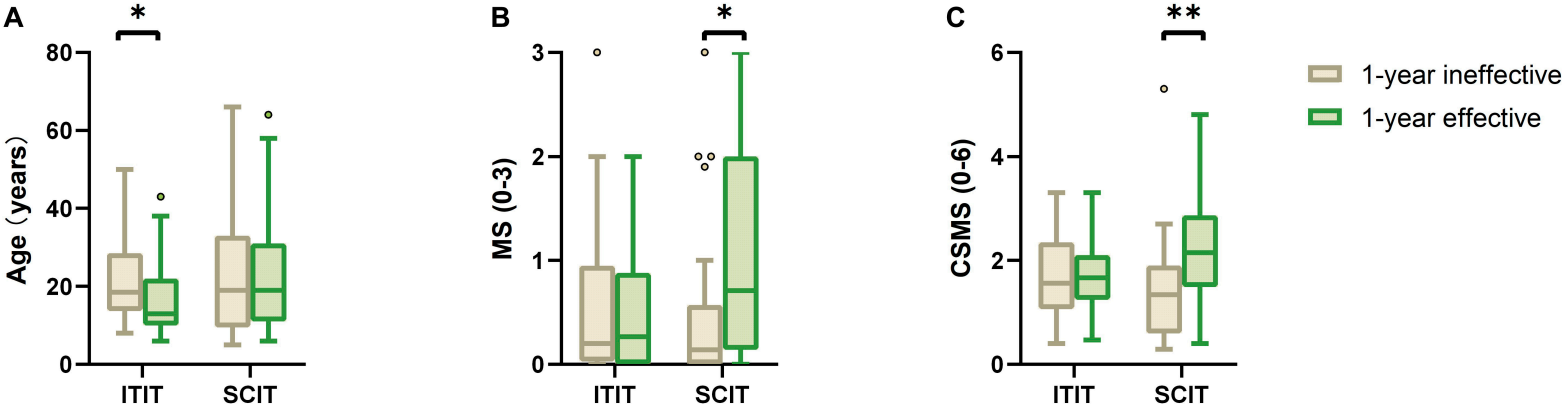
Influence factors of ITIT and SCIT. Patients were categorized into effective and ineffective groups based on TI at 1 year. The effects of (A) age, (B) MS, and (C) CSMS before treatment on the 2 groups of patients are indicated. **P < 0.01 and *P < 0.05.

To identify factors influencing 1-year efficacy, we divided patients into effective and ineffective groups based on whether their TI was greater than 33% at the 1-year follow-up, and compared clinical characteristics and immune parameters at baseline between the subgroups. The results revealed that younger individuals responded more favorably to ITIT, while no such age-related differences were observed in SCIT outcomes (Fig. 5A). Severe pretreatment symptoms and higher medication usage correlated with greater improvement after SCIT, but this was not seen in ITIT (Fig. 5B and C). No statistical differences in immune markers were found between the subgroups at baseline, and detailed data were presented in Table 2.

**Table 2.** The characteristics and laboratory findings at baseline of the SCIT and ITIT patients with different outcomes

Characteristic		SCIT				ITIT			
		1-year ineffective	1-year effective	Total	*P* value	1-year ineffective	1-year effective	Total	*P* value
Number of patients		29	31	60		22	38	60	
Female sex, *n* (%)		9 (31.03)	14 (45.16)	23 (38.33)	0.261	7 (31.82)	17 (44.74)	24 (40.00)	0.325
Age (years), median (IQR)		19.00 (9.50–33.00)	19.00 (11.00–31.50)	19.00 (10.00–31.75)	0.678	17.00 (14.00–40.00)	13.50 (10.00–22.75)	15.00 (11.00–24.75)	0.038
Age group, *n* (%)	≤18	14 (48.28)	15 (48.39)	29 (48.33)	0.993	11 (50.00)	26 (68.42)	37 (61.67)	0.157
	≤14	13 (44.83)	12 (38.71)	25 (41.67)	0.631	7 (31.82)	22 (57.89)	29 (48.33)	0.050
	≤13	13 (44.83)	11 (35.48)	24 (40.00)	0.460	5 (22.73)	20 (52.63)	25 (41.67)	0.024
	≤12	12 (41.38)	9 (29.03)	21 (35.00)	0.316	4 (18.18)	17 (44.74)	21 (35.00)	0.038
	≤11	11 (37.93)	9 (29.03)	20 (33.33)	0.465	2 (9.09)	14 (36.84)	16 (26.67)	0.019
CSMS, mean (SD)		1.46 (1.05)	2.22 (1.07)	1.86 (1.07)	0.007	1.70 (0.81)	1.66 (0.60)	1.68 (0.67)	0.899
SS, mean (SD)		1.02 (0.67)	1.25 (0.66)	1.13 (0.67)	0.197	1.09 (0.54)	1.30 (0.56)	1.26 (0.54)	0.235
MS, median (IQR)		0.14 (0.00–0.57)	0.71 (0.14–2.00)	0.29 (0.00–1.07)	0.022	0.20 (0.03–0.93)	0.27 (0.00–0.95)	0.20 (0.00–0.93)	0.470
VAS, mean (SD)		17.80 (11.74)	22.84 (11.66)	20.18 (11.68)	0.100	19.32 (9.50)	23.24 (10.22)	22.36 (9.74)	0.228
Family history of atopy, *n* (%)		4 (13.79)	8 (25.81)	12 (20.00)	0.401	7 (31.82)	10 (26.32)	17 (28.33)	0.649
History of drug allergy, *n* (%)		5 (17.24)	8 (25.81)	13 (21.67)	0.421	4 (18.18)	5 (13.16)	9 (15.00)	0.599
Monosensitization to dust mites, *n* (%)		21 (72.41)	20 (64.52)	41 (68.33)	0.511	16 (72.73)	22 (57.89)	38 (63.33)	0.251
tIgE (pg/ml), median (IQR)		230.00 (73.60–750.50)	348.00 (236.50–636.75)	316.00 (109.00–706.00)	0.142	375.50 (238.50–814.25)	392.00 (175.00–1060.00)	387.50 (180.25–849.00)	1.000
Der p-sIgE (kU/l), median (IQR)		16.07 (5.54–45.08)	22.96 (3.31–50.76)	19.86 (4.22–47.96)	0.970	27.41 (6.48–100.00)	48.82 (10.77–100.00)	45.14 (8.31–100.00)	0.775
Der f-sIgE (kU/l), median (IQR)		15.49 (8.41–51.75)	20.73 (6.66–81.13)	20.67 (6.17–73.89)	0.821	77.03 (14.73–100.00)	100.00 (17.36–100.00)	100.00 (17.08–100.00)	0.589
Dust mite-sIgE (kU/l), median (IQR)		30.98 (14.92–103.49)	43.87 (12.24–120.18)	34.91 (13.13–109.38)	0.706	103.21 (21.70–200.00)	145.04 (33.58–200.00)	141.47 (26.80–200.00)	0.838
Dust mite-sIgE/tIgE, median (IQR)		0.08 (0.07–0.19)	0.19 (0.05–0.34)	0.16 (0.06–0.30)	0.533	0.23 (0.07–0.37)	0.23 (0.06–0.56)	0.23 (0.06–0.48)	0.912
EOS%, median (IQR)		4.20 (2.70–8.20)	4.90 (2.75–8.35)	4.45 (2.73–8.28)	0.786	3.60 (2.70–7.10)	6.10 (4.30–8.88)	5.40 (2.80–8.30)	0.079
BAS%, median (IQR)		0.30 (0.20–0.80)	0.40 (0.25–0.50)	0.30 (0.20–0.70)	0.781	0.40 (0.30–0.70)	0.60 (0.40–0.90)	0.45 (0.30–0.80)	0.209
IgG, mean (SD)		11.22 (1.76)	11.73 (2.03)	11.24 (1.96)	0.117	12.0826 (2.61108)	11.7329 (2.55179)	11.85 (2.55)	0.670
IgA, mean (SD)		2.25 (0.53)	2.49 (1.17)	2.38 (0.95)	0.469	2.1311 (1.02240)	1.9703 (0.75596)	2.03 (0.85)	0.472
IL-2, median (IQR)		4.91 (4.16–6.21)	4.71 (3.65–6.20)	4.81 (3.90–6.21)	0.580	2.43 (2.32–2.81)	2.46 (2.20–2.85)	2.46 (2.29–2.85)	0.613
IL-4, median (IQR)		4.74 (3.56–5.42)	4.76 (4.01–5.78)	4.74 (3.76–5.58)	0.428	3.83 (3.49–4.39)	3.53 (3.12–4.14)	3.70 (3.27–4.29)	0.482
IL-5, mean (SD)		26.36 (11.72)	30.50 (10.33)	28.70 (10.90)	0.378	28.60 (3.87)	27.08 (5.12)	28.41 (1.40)	0.547
IL-6, median (IQR)		7.68 (5.37–13.16)	7.42 (5.42–13.72)	7.51 (5.43–13.16)	0.854	5.10 (4.42–9.57)	5.31 (4.43–10.87)	5.10 (4.45–10.17)	0.934
IL-10, median (IQR)		4.93 (4.12–5.91)	4.70 (3.98–5.33)	4.78 (4.00–5.35)	0.672	3.84 (3.44–4.66)	3.67 (3.26–4.40)	3.69 (3.30–4.40)	0.365
IL-13, median (IQR)		18.00 (17.00–19.00)	25.00 (17.50–30.00)	18.27 (17.47–25.63)	0.196	20.77 (15.24–23.06)	21.62 (18.08–23.97)	21.61 (16.23–23.26)	0.631
IL-17, median (IQR)		5.79 (2.67–8.72)	2.63 (2.33–2.95)	2.77 (2.33–5.47)	0.186	2.19 (1.95–2.70)	2.15 (1.86–2.54)	2.15 (1.88–2.55)	0.487
TNF-α, median (IQR)		4.90 (2.70–8.65)	3.27 (2.78–3.96)	3.45 (2.76–5.36)	0.294	3.33 (2.59–4.36)	3.49 (2.85–6.01)	3.49 (2.75–5.67)	0.546
IFN-γ, median (IQR)		2.83 (2.53–3.59)	2.99 (2.68–3.41)	2.97 (2.69–3.43)	0.685	2.25 (1.92–2.87)	2.25 (2.01–2.54)	2.25 (2.01–2.57)	0.875

### Safety profile

**Fig. 6.**
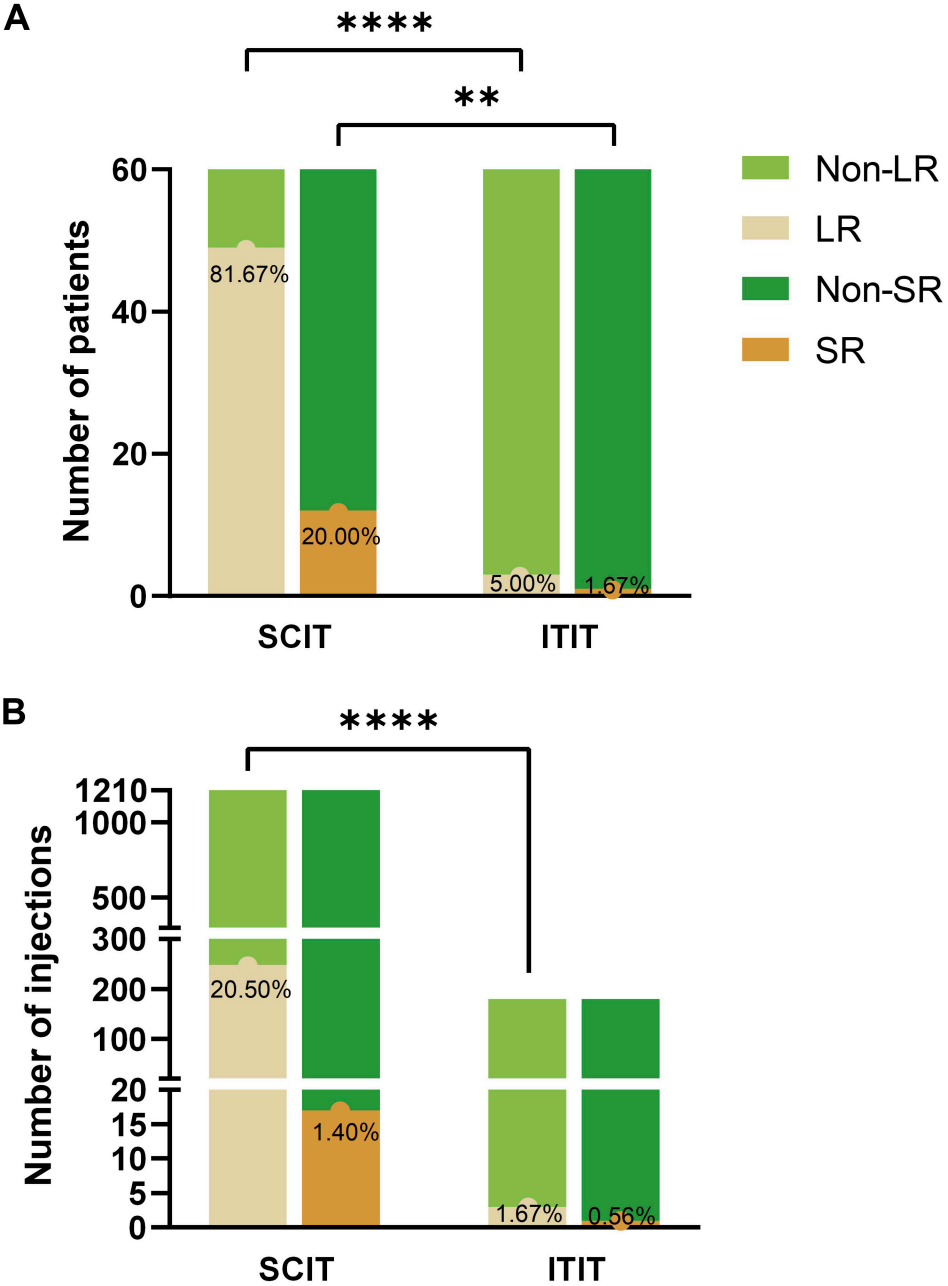
Adverse reactions in the 2 groups during the first year of treatment. (A) Number of patients experiencing adverse reactions. (B) Number of injections associated with adverse reactions. ****P < 0.0001 and **P < 0.01. LR, local reactions; SR, systemic reactions.

ITIT showed significantly fewer adverse reactions compared to SCIT. In the SCIT group, 20.50% of injections in 49 patients (81.67%) led to local reactions (LRs), while 1.40% of injections in 12 patients (20.00%) caused systemic reactions (SRs). In contrast, only 3 ITIT patients (5.00%) experienced tonsil pain or enlargement, and 1 patient (1.67%) developed diarrhea following injection (Fig. 6). The incidence of local adverse reactions was significantly lower in ITIT, and fewer patients experienced systemic adverse reactions following intratonsillar injections. Detailed data for both groups are presented in Table 3.

**Table 3.** Adverse reactions in SCIT and ITIT groups

	Number of LRs (%)	Number of SRs (%)	Patients with LRs (%)	Patients with SRs (%)
SCIT	248 (20.50%)	17 (1.40%)	49 (81.67%)	12 (20.00%)
ITIT	3 (1.67%)	1 (0.56 %)	3 (5.00%)	1 (1.67%)
χ^2^ value	36.2821	0.3447	68.7217	8.6269
*P* value	<0.0001	0.5571	<0.0001	0.0033

### Predictors of 1-year efficacy

**Fig. 7.**
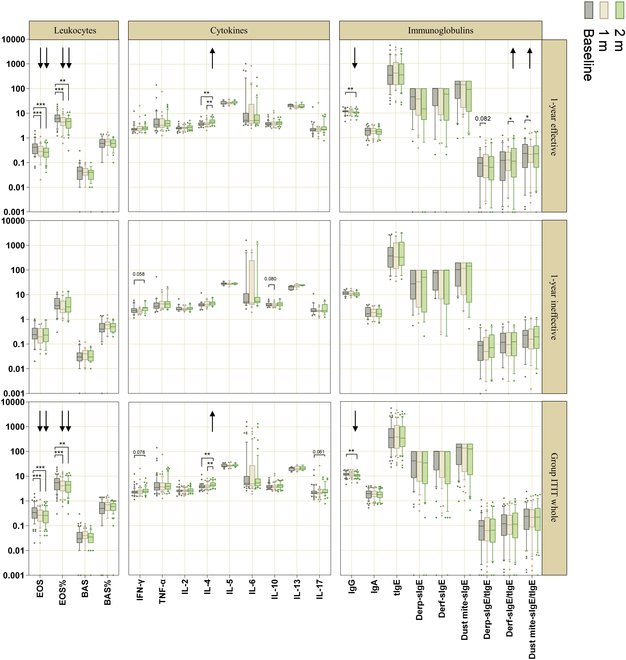
Box plots illustrating changes in immune markers in peripheral blood of ITIT patients over time. The left *y* axis is presented on a log_10_ scale, while the right *y* axis indicates patient groups. Arrows indicate significant decrement (downward) or increment (upward) of immunological parameters after treatment. ***P < 0.001, **P < 0.01, and *P < 0.05. EOS, eosinophil granulocyte; BAS, basophilic granulocyte; IFN, interferon; TNF, tumor necrosis factor; IL, interleukin; Ig, immunoglobulin; tIgE, total IgE; sIgE, specific IgE. Dust mite-sIgE = Der p-sIgE + Der f-sIgE. Units: EOS, BAS: 10 × 9/l; EOS%, BAS%: %; cytokines: pg/ml; IgG, IgA: g/l; tIgE, Der p-sIgE, Der f-sIgE, dust mite-sIgE: kU/l; Der p-sIgE/tIgE, Der f-sIgE/tIgE, dust mite-sIgE/tIgE: unitless.

Based on the baseline data in Table 2, no immunological parameters at baseline were identified that influenced the 1-year efficacy of ITIT. Early (within 2 months) changes in eosinophil (EOS), IL-4, and sIgE/tIgE ratios predicted 1-year efficacy in ITIT. Decreases in EOS absolute value and percentage, along with increases in IL-4 at months 1 and 2, were observed. Significant increases in *Dermatophagoides pteronyssinus* (Der p)-sIgE/tIgE and *Dermatophagoides farinae* (Der f)-sIgE/tIgE levels were also noted, indicating early immunological shifts toward efficacy (Fig. 7). In the SCIT group, a comparable pattern was observed, with reductions in EOS, alongside increases in IL-4 and tIgE levels in 2 months (Fig. S1). These results highlight similar early immune responses in both ITIT and SCIT.

### T cell subset dynamics

**Fig. 8.**
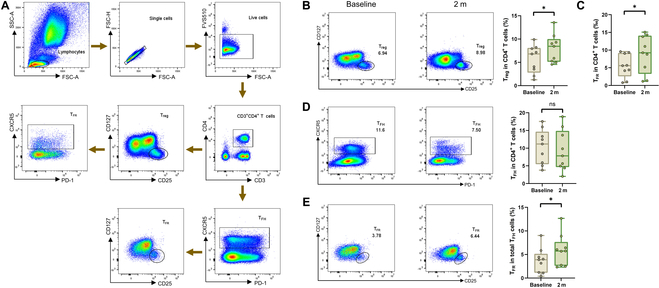
Frequencies of T cells before and after ITIT. PBMCs from ITIT individuals (baseline and month 2) were stained with a panel of antibodies and analyzed by flow cytometry. (A) Gating strategy. Within live CD3^+^CD4^+^ T cells, regulatory T (T_reg_) cells were defined as CD25^+high^CD127^low^ cells, follicular regulatory T (T_FR_) cells were defined as CXCR5^+^CD25^+high^CD127^low^ cells, and follicular helper T (T_FH_) cells were defined as CXCR5^+^ cells. Frequencies of (B) T_reg_ cells, (C) T_FR_ cells, and (D) T_FH_ cells in CD3^+^CD4^+^ T cells are shown. Frequencies of (E) T_FR_ cells in T_FH_ cells are shown, too. *P < 0.05. ns represents *P* > 0.05.

To further explore the cellular and molecular mechanisms underlying the immune tolerance induced by ITIT, we analyzed changes in the proportions of circulating T cell subsets, including regulatory T (T_reg_), follicular regulatory T (T_FR_), and T_FH_ cells. The gating strategy is illustrated in Fig. 8A. ITIT induced an increase in circulating T_reg_ and T_FR_ cells within 2 months of treatment, although T_FH_ cell frequencies remained unchanged (Fig. 8B to D). The T_FR_/T_FH_ ratio showed a significant increase, suggesting a regulatory immune shift in response to ITIT (Fig. 8E).

## Discussion

Vaccines administered through traditional routes must reach secondary lymphoid organs to effectively induce an immune response [21]. Compared to subcutaneous and intradermal administration, direct injection into lymph nodes enhances T cell responses to antigens, greatly increasing immunogenicity [22]. Clinical studies showed that ILIT can rapidly induce immune tolerance and reduce the risk of systemic allergic reactions [4,6–9,12,23]. However, precise injection techniques are required due to the proximity of lymph nodes (inguinal and cervical) to important structures [11]. Even with ultrasound guidance, there remains a risk of unintentional injection into surrounding tissues, such as blood vessels [24]. Moreover, lymph nodes connect to systemic circulation via the thoracic duct, posing potential risks of severe adverse events. One study reported moderate-to-severe SRs in up to 15% of ILIT injections, even at low concentrations [25].

The tonsils are secondary lymphoid organs located at the lateral wall of the oropharynx, intricately connected to the mucosa and lymphoid tissues surrounding the nasal cavity, establishing a close relationship with the nasal cavity [14]. Unlike lymph nodes, tonsils can directly receive allergen stimulation from areas such as the nose, throat, and oral cavity, participate in immune responses of the respiratory tract, and generate immune memory. Theoretically, allergens can be transported to the tonsils via nasal secretions and the wiggle of nasal cilia, stimulating primary lymphoid follicles, forming germinal centers, and activating B lymphocytes. This process generates plasma cells that produce sIgE, which are absorbed by mast cells and transported in the nasal mucosa [26].

We did not detect dust mite-sIgE in the tonsils of non-atopic patients, while dust mite-sIgE was positive in the tonsils of the majority (80%) of AR patients. Additionally, tonsillar tIgE and dust mite-sIgE levels were significantly higher than those in peripheral blood and positively correlated with peripheral blood IgE levels (Fig. 1C to J). These results further confirm the involvement of tonsils in IgE-mediated sensitization, underscoring their importance in allergen recognition and immune response.

T_FH_ cell subsets and functional molecules (primarily IL-4 and IL-21) play critical roles in the occurrence, progression, and maintenance of allergic diseases [27]. IL-4 can promote T_H_2 cell differentiation, increase the release of inflammatory mediators, and induce IgE production [28]. IL-21, a hallmark cytokine of T_FH_ cells, also plays an important role in T_FH_ cell generation and germinal center formation, contributing to the formation of immune memory against allergens [29–31]. Previous studies have shown higher levels of T_FH_ cells in the peripheral circulation of AR patients [32]. This study found that the levels of IL-4 and IL-21 in the tonsils of AR patients were significantly elevated (Fig. 1A and B), which may lead to increased sensitivity to allergens and increased IgE production, indicating that the tonsils, as immune organs, play important regulatory and maintenance roles in the occurrence and progression of allergic diseases. Consequently, allergen administration directly into the tonsils may induce faster and longer-lasting efficacy by altering the immune status of the tonsils. Compared to ILIT, which requires ultrasound-guided injection into deep lymph nodes, this approach is more convenient and carries lower risks; meanwhile, it can leverage the advantages of adjacency to promote local immune responses. Importantly, the persistence of immune activity in adult tonsils suggests the potential efficacy of tonsillar injection-based AIT, with stronger responses observed in younger individuals, which may further enhance treatment outcomes (Fig. 1A to E).

In our 1-year clinical follow-up, ITIT demonstrated significant efficacy, particularly with rapid symptom relief early in treatment. ITIT was superior to SCIT in maintaining effectiveness from 1 to 6 months after treatment (Fig. 3). Comparison of efficacy between the ITIT and SCIT groups at different time points consistently showed higher effectiveness in the ITIT group, with fewer instances of delayed onset or ineffectiveness (Fig. 4). This suggests that ITIT offers more stable and enduring efficacy in 1 year. Stratifying by age revealed better outcomes in younger patients (≤14 years) than older individuals (Fig. 5 and Table 2). This may be due to greater immune plasticity and the effect of age on tonsillar structure and function [33–36], which is consistent with our previous tests on the tonsils (Fig. 1). Early tonsil injections may be more effective at reversing allergic reactions in younger patients.

In addition to differences in injection sites, the main distinctions between SCIT and ITIT lie in dosage and injection frequency. The conventional SCIT regimen typically starts with a dose of 5 therapeutic units (TU), increasing weekly until a maintenance dose of 5,000 TU is reached. In contrast, our ITIT protocol, based on the ILIT schedule, involves only 3 injections over 2 months [4]. This approach minimizes immune system overstimulation and is also in line with vaccine immunology principles [36]. We selected an initial dose of 5 TU, which effectively alleviated symptoms without causing adverse reactions. Direct antigen administration into lymphoid tissue is known to be 100 to 1,000 times more efficient than intradermal routes [21]. ILIT has been shown to induce allergen-specific peripheral tolerance with a cumulative allergen dose 1,000 times lower than that used in traditional immunotherapy over 3 years [4,8]. Thus, we employed a maintenance dose of 50 TU for the remaining injections. Compared to SCIT, ITIT achieves equivalent clinical efficacy with ^1^/_500_ of the allergen exposure over a 1-year period.

Furthermore, unlike the dermis, tonsils contain few mast cells and act as immune organs with regulatory capabilities against external immune stimuli, thereby reducing the risk of adverse effects from immune stimulation [33,35,37]. In contrast, injecting drugs and allergens into lymph tissues, which are deep within the body, may provoke a broader immune response. A previous study showed that among 58 patients receiving inguinal lymph node injections, 6 experienced mild systemic allergic reactions such as urticaria and angioedema [4]. Another study reported that 3 of 12 patients receiving ILIT had mild to moderate lymph node enlargement, 1 had mild nausea, 1 had mild respiratory distress, and 1 had a moderate rash [6]. Our study showed only 3 cases of mild tonsillar pain and one case of mild diarrhea among the 60 ITIT patients, with no moderate or severe adverse events (Table 3). This safety profile is comparable to, or even better than, ILIT and significantly safer than SCIT (Fig. 6). The occurrence of diarrhea may have been due to accidental drug leakage into the gastrointestinal tract, highlighting the importance of precise injection techniques. It is also worth noting that upper respiratory infections could sensitize the tonsils, increasing the likelihood of LRs; therefore, ITIT treatment should be postponed until such infections subside to ensure optimal safety and efficacy.

In a recent published ITIT study, AR patients aged 18 to 55 received 6 house dust mite (HDM) (Alutard SQ; ALK, Hørsholm, Denmark) injections over 3 months. Compared with placebo controls, ITIT began to show effectiveness at least 3 months after treatment, with uncertain improvements at 6 and 12 months [38]. In contrast, our study included a younger cohort (5 to 17 years), and we found that younger patients experienced better outcomes, suggesting that age may significantly impact treatment effectiveness. The 4-week injection interval we used may have contributed to better immune response development, as shorter intervals may hinder the formation of high-affinity B and T cell responses [24,36]. Additionally, the total HDM allergen dose in the other study was 27 times higher than in our protocol, indicating that optimizing both dosage and schedule is crucial for maximizing treatment outcomes [39].

We further analyzed immunological markers associated with treatment efficacy. EOSs are important markers of allergic inflammation [40,41], and we observed that patients with better 1-year efficacy showed early reductions in EOS count and percentage, reflecting the regulatory effect of the immune system and the improvement in inflammation severity, which may indicate long-term effectiveness. AIT typically reduces IL-4, IL-5, and IL-13 produced by T_H_2 cells while increasing interferon-γ (IFN-γ) from T_H_1 cells [42]. However, AIT’s transition from a T_H_2- to a T_H_1-dominant response is often delayed [43,44]. In our study, IL-4 levels increased during early AIT treatment (Fig. 7 and Fig. S1), consistent with reports of elevated T_H_2 cytokines in the early stages of AIT [45,46]. This early rise in IL-4 likely reflects a germinal center response in lymphoid tissue [47]. In contrast, IL-13 primarily affects structural cells, and IL-5 plays a key role in EOS differentiation and maturation [48–50]. We did not observe early changes in IL-5 or IL-13 (Fig. 7 and Fig. S1), possibly because their effects in allergen-specific T cells have been diluted by other peripheral immune responses [44]. These findings emphasize the role of IL-4 as a sensitive marker of early immune modulation, while IL-5 and IL-13 are likely to reflect immune resolution and remodeling in later stages of treatment.

The allergen specificity of immunotherapy is crucial for understanding its benefits and potential mechanisms. Successful AIT is characterized by an early increase in serum-sIgE/tIgE [44]. In this study, patients who showed better 1-year efficacy also experienced early increases in Der p-sIgE/tIgE and Der f-sIgE/tIgE levels within the first 2 months of ITIT treatment (Fig. 7). This early IgE response may be due to heightened IL-4 production, while the initial tIgE increase observed with SCIT likely reflects a broader immune activation (Fig. S1). Additionally, there was a decrease in serum IgG levels at the 2-month mark (Fig. 7), suggesting that measuring allergen-specific IgG or IgG subclasses (including IgG1 and IgG4) blocking activity may be more informative in evaluating treatment efficacy.

T_reg_ cells play a crucial role in maintaining peripheral tolerance [42]. T_FR_ cells, defined as CXCR5^+^ T_reg_ cells, can migrate to germinal centers to regulate T and B cell responses, making them a distinct subset of regulatory CD4 T cells [51,52]. The imbalance in the T_FH_-T_FR_ cell regulatory axis may be a common feature of allergic diseases, which can be restored by AIT [31]. ITIT treatment led to a significant increase in both T_reg_ and T_FR_ cell frequencies, along with a rise in the T_FR_/T_FH_ ratio (Fig. 8). This change may represent the regulatory effect of ITIT on the immune system and the restoration of immune balance, thereby helping to alleviate symptoms and inflammatory responses in allergic diseases.

In brief, this is the first report comparing ITIT to traditional immunotherapy as a new injecting route of AIT. ITIT offers a less invasive alternative to SCIT, with fewer adverse effects and reduced treatment complexity and cost. This will allow more AR patients, particularly those with time constraints, to benefit from immunotherapy sooner, thereby reducing the disease burden.

However, this study has several limitations. For ethical reasons, we were unable to collect posttreatment tonsil tissue samples to observe immunological changes. Furthermore, the trial lacked a placebo-controlled design and blinding. Conducted as an open-label trial, the study inherently carries risks of bias, including observer expectations and reliance on patient self-reporting. Additionally, for this new treatment, we did not strictly explore the optimal dosing regimen, and the follow-up period was limited to 1 year, with insufficient investigation into the underlying mechanisms.

The reason we chose SCIT rather than placebo as a control is that when a standard treatment is available, ethics often require new therapies to be compared against this standard. There are notable differences in both the administration site and the number of needles of ITIT and SCIT, so the blind method is not feasible. To minimize bias, the results were assessed by independent blinded evaluators, and the data were anonymized for unbiased statistical analysis. Despite these limitations, our findings suggest that ITIT holds promise as a viable alternative to conventional SCIT, warranting further investigation and broader clinical application. Future clinical trials should include longer follow-up periods, higher-quality designs, and a more rigorous exploration of the optimal allergen dosage for ITIT. These studies should also investigate the long-term efficacy of ITIT and whether booster shots are necessary to maintain treatment effects. Furthermore, advanced techniques such as single-cell sequencing and proteomics could be employed to gain deeper insights into the therapeutic mechanisms of ITIT.

## Conclusion

This study is the first to compare ITIT with traditional immunotherapy, demonstrating its potential as a convenient, effective, and safer AIT alternative. This new technique markedly reduces injection frequency and allergen dosage while maintaining safety and enhancing treatment compliance, making it a promising option for AR treatment. Future research should focus on optimizing dosage and injection schedules, exploring personalized treatment strategies, and evaluating long-term efficacy. Investigating the mechanisms underlying ITIT will provide further insights into its therapeutic potential and pave the way for broader clinical application.

## Materials and Methods

### Palatine tonsil samples

#### Tissue collection

Tonsil specimens were obtained from patients undergoing tonsillectomy due to tonsillar hypertrophy, with or without dust mite-induced AR, to examine the tonsil’s role in immune responses associated with nasal allergy. Samples showing signs of infection or microabscess were excluded. The study was conducted in accordance with the ethical guidelines and approved by the Ethics Committee of Renmin Hospital, Wuhan University (approval no. WDRY2018-K020).

#### Assessment of immune status

Regions of severe clotting or cauterization in the tonsil tissue were first removed, followed by washing with precooled phosphate-buffered saline (PBS) to remove blood. The tissue was then weighed and dissected into small pieces. The tissue pieces were homogenized in precooled PBS at a ratio of 9:1 (PBS to tissue) using a liquid nitrogen grinder (Retsch MM 400, Hann, Germany) for 10 min. The homogenate was centrifuged at 5,000 rpm for 5 min at 4 °C, and the supernatant was collected.

The concentrations of IL-4, IL-21, and total IgE (tIgE) in the tonsil tissue homogenate were assessed using sandwich enzyme-linked immunosorbent assay (ELISA) kits (R&D Systems, MN, USA) according to the manufacturer’s instructions. The supernatant was added to precoated plates with human IL-4, IL-21, or tIgE monoclonal antibodies and incubated at 37 °C for 2 h. After thorough washing, horseradish peroxidase (HRP)-conjugated detection antibodies were added, followed by incubation and washing. The substrate tetramethylbenzidine (TMB) was then added for color development, and the absorbance was measured at 450 nm using an EnSight multimode plate reader (PerkinElmer, Waltham, MS, USA) to determine the cytokine concentrations (pg/ml). All samples were analyzed in duplicate, and the mean values were used for statistical analysis.

The levels of dust mite-specific IgE (sIgE) in the tonsil tissue homogenate were measured using a microfluidic-based automated immunoassay system (BioIC) (Agnitio, Taiwan, China). Following the manufacturer’s instructions, the chip was loaded with 100 μl of sample, 450 μl of washing buffer, 120 μl of premixed substrate, and 120 μl of HRP conjugate diluted at 1:1,000. Subsequently, the chip was inserted into the BioIC instrument (Agnitio, Taiwan, China) for automated chemiluminescent immunoassay, specifically detecting sIgE for allergens.

### Clinical trial design

The study was crafted as a single-center, prospective, open-label, positive parallel-controlled intervention trial. Patient grouping was carefully tailored to individual conditions and preferences to enhance clinical feasibility. The primary objective was to comprehensively compare the efficacy and safety of ITIT against SCIT in patients diagnosed with AR. The study protocol has been reviewed and approved by the Ethics Committee of Renmin Hospital of Wuhan University, permission numbers 2021-1-X-2 and 2024-K097. It was conducted with the written informed consent of all participants.

### Clinical trial patients

Patients with dust mite-induced AR were recruited from the Department of Rhinology and Allergy of Renmin Hospital of Wuhan University, between August 2021 and September 2022. A total of 120 subjects were included after comprehensive evaluation.

Inclusion criteria followed the Allergic Rhinitis and its Impact on Asthma (ARIA) guidelines [53] and required patients to be aged 5 to 65 years with moderate to severe dust mite-induced AR. Diagnostic criteria included (a) persistent nasal symptoms such as rhinorrhea, sneezing, and nasal congestion and (b) confirmed dust mite allergy via SPT and serum sIgE levels. SPT-positive reactions were graded using the skin index (SI), with SI representing the ratio of the mean diameter of allergen wheals to the mean diameter of histamine wheals, categorized as follows: SI < 0.3, −; 0.3 to 0.5, +; 0.5 to 1, ++; 1 to 2, +++; SI ≥ 2, ++++. SIgE values were categorized into 7 grades: sIgE < 0.35 kU/l, grade 0 (negative); 0.35 to 0.7 kU/l, grade 1; 0.7 to 3.5 kU/l, grade 2; 3.5 to 17.5 kU/l, grade 3; 17.5 to 50 kU/l, grade 4; 50 to 100 kU/l, grade 5; sIgE ≥ 100 kU/l, grade 6. It is required positivity for Der p and Der f (SPT results of ++ or higher and/or serum sIgE ≥ grade 2), and negativity for other allergens in allergen testing.

Exclusion criteria included severe or uncontrolled asthma [forced expiratory volume in 1 s (FEV1) < 70% of predicted] and irreversible obstructive airway disease, current treatment with β-blockers or angiotensin-converting enzyme inhibitors (ACEi), severe cardiovascular or autoimmune diseases, a history of severe recurrent acute or chronic sinusitis, severe liver or kidney damage, severe psychiatric disorders, malignancy, pregnancy or plans to become pregnant, immunotherapy within the past 3 years, treatment with IgE monoclonal antibodies in the past 4 months, experimental drug use in the past 30 d, enlarged tonsils or chronic tonsillitis, atrophic tonsils or tonsils have been removed, inability to understand trial risks, or any medical condition deemed incompatible with trial participation by the investigators.

### Immunization protocols

#### Run-in treatment

After signing informed consent, eligible participants were entered into a run-in period. During this period, all participants were administered intranasal corticosteroids and oral antihistamines as needed for 1 week. The intranasal corticosteroid spray was administered twice daily at a dose of 128 μg per application (64 μg per nostril). The oral antihistamine was given once daily at a 10-mg dose. Participants were required to complete this 7-d run-in treatment with at least 80% adherence to qualify for the treatment phase.

#### ITIT group

Patients in the ITIT group received 3 intratonsillar injections of a standardized dust mite allergen extract (Novo Helisen-Depot, Allergopharma, Reinbek, Germany) every 4 weeks. The allergen extract is a mixture of Der p and Der f, adsorbed onto aluminum hydroxide for allergen immunotherapy. The extract came in 3 vials with allergen concentrations of 50, 500, and 5,000 TU/ml. The first injection used 0.1 ml from vial 1 (5 TU), while the second and third injections used 0.1 ml from vial 2 (50 TU). The cumulative dose of HDM allergen used for ITIT was 105 TU, corresponding to 0.1344 μg of Der p and Der f.

Prior to each injection, patients were administered one tablet of antihistamine.

After injection, vital signs and peak expiratory flow were monitored for 30 min in the hospital. Adverse reactions, if any, were managed and documented. Patients received oral antihistamines and nasal corticosteroids as needed and had follow-up appointments at 1, 2, 3, 6, and 12 months after treatment initiation. Symptom data were also collected via WeChat between appointments.

#### SCIT group

SCIT was administered in 2 stages: accumulation and maintenance. During the first 14 weeks, patients received a series of subcutaneous injections, starting with 0.1 ml from the first vial and gradually increasing to 1.0 ml from the third vial. The maintenance dose was 5,000 TU administered every 4 to 6 weeks. The total cumulative HDM dose for SCIT was 66,325 TU, equivalent to 84.896 μg of Der p and Der f. Injection protocols and monitoring procedures were similar to those of the ITIT group.

### Outcome measures

#### Primary endpoint

1. CSMS improvement: The primary endpoint was improvement in CSMS, which tracks changes in symptoms and medication use to evaluate treatment sustainability and effectiveness. CSMS includes the following: (a) SS: Allergic symptoms such as nasal congestion, rhinorrhea, nasal itching, sneezing, eye itching, and tearing were scored from 0 (no symptoms) to 3 (severe symptoms). The average of these scores constituted the SS. (b) MS: Points were assigned based on daily use of antihistamines (1 point), nasal corticosteroids (2 points), or oral corticosteroids (3 points). The highest score of the day was recorded as the MS. CSMS (0 to 6) = SS (0 to 3) + MS (0 to 3).

CSMSs were assessed at baseline and at 1, 2, 3, 6, and 12 months after treatment initiation. Improvement was analyzed over time using distinct grouping strategies tailored to specific research objectives:

TI stratification: Patients were stratified into 5 groups based on the TI, calculated as follows: TI (%) = (pretreatment CSMS − posttreatment CSMS)/pretreatment CSMS × 100%. The 5 categories are as follows: symptomless (TI = 100%), markedly effective (TI > 66%), effective (TI > 33%), poorly effective (TI 0 to 33%), and nonresponsive (TI < 0). This analysis provides insights into treatment efficacy at different follow-up time points.

Improvement duration analysis: The improvement in CSMS for each patient was then analyzed, categorizing patients into 4 groups based on the duration of improvement: (a) consistently effective: patients with clinically significant improvement (TI > 33%) within the first 3 months of treatment, maintaining a stable response throughout the follow-up period; (b) delayed-term effective: patients whose clinically significant improvement (TI > 33%) was observed only after 3 months of treatment; (c) short-term effective: patients who showed clinically significant improvement (TI > 33%) within the first 3 months but experienced a loss of efficacy (falling below the 33% threshold) at later follow-ups; (d) ineffective: patients who did not maintain TI > 33% at both early and late follow-ups.

These strategies are designed to comprehensively assess the dynamic nature of symptom improvement, enabling a detailed analysis of the treatment characteristics of ITIT and SCIT.

2. Adverse reactions: Patients were observed for 30 min after injection and were encouraged to report any adverse events during follow-up. Adverse reactions were classified as LRs or SRs according to the World Allergy Organization’s Subcutaneous Immunotherapy Response Classification System. All reactions were documented in detail, including timing, injection number, symptoms, and management.

#### Secondary endpoint

1. VAS improvement: Patients rated symptom severity on a scale from 0 (asymptomatic) to 10 (most severe) during CSMS assessments. A total score, ranging from 0 to 60, was aggregated from evaluations of nasal congestion, rhinorrhea, itching, and sneezing at baseline and after 1, 2, 3, 6, and 12 months.

2. Changes in immune markers

a. Blood leukocytes: Changes in blood EOS and basophil (BAS) counts and percentages were evaluated before treatment and at 1- and 2-month posttreatment intervals.

b. Key serum cytokines: Levels of cytokines such as tumor necrosis factor-α (TNF-α), IFN-γ, IL-2, IL-4, IL-5, IL-6, IL-10, IL-13, and IL-17 were measured at the same time points.

c. Serum immunoglobulins: TIgE, IgA, IgG, and dust mite-sIgE levels were analyzed at the same intervals as above.

#### Exploratory indicators: Changes in T cell differentiation ratio

Flow cytometry was used to assess peripheral blood T cell subsets, including T_reg_ cells, T_FR_ cells, and T_FH_ cells, before and 1 and 2 months after ITIT. Peripheral blood mononuclear cells (PBMCs) were isolated using mononuclear cell separation solution (TBD Science, Tianjin, China), and surface marker staining was performed using antibodies such as anti-CD3-APC-Cy7, anti-CD4-BB515, anti-CXCR5-Alexa Fluor 647, anti-PD-1-PE, anti-CD25-BV421, and anti-CD127-PE-Cy7 (BD Biosciences, San Jose, CA, USA). Flow cytometry analysis was conducted on a BD FACSCalibur flow cytometer (BD Biosciences), and stained cells were analyzed using FlowJo software (version 10.8.1; TreeStar, Ashland, OR, USA).

### Statistical analysis

Statistical analyses were conducted using SPSS version 26 (IBM, Endicott, NY, USA). Normality of data distribution was assessed via the D’Agostino–Pearson comprehensive normality test. Normally distributed data were reported as mean ± SD or SEM, whereas nonnormally distributed data were expressed as median with interquartile range (IQR). Paired *t* tests and Wilcoxon signed-rank tests were used for within-group comparisons of normally and nonnormally distributed continuous variables, respectively. Between-group comparisons involved unpaired *t* tests or Mann–Whitney *U* tests for normally and nonnormally distributed continuous variables, respectively. Categorical variables, presented as *n* (%), were analyzed via Fisher’s exact test or Pearson’s chi-square test. Pearson correlation analysis examined correlations involving sIgE values, and Spearman rank correlation was used for correlations between SPT and sIgE rank. The analysis of covariance (ANCOVA) was performed to adjust for baseline dust mite-sIgE levels and to assess the significance of covariate effects. For nonnormally distributed variables, natural logarithmic transformations were applied to achieve normality prior to ANCOVA. *P* values were 2-tailed, with significance set at *P* < 0.05.

## Ethical Approval

The study was done in accordance with the Declaration of Helsinki and the Good Clinical Practice guidelines. The protocol and all amendments were approved by the Ethics Committee of Renmin Hospital of Wuhan University (approval numbers: 2018-K020, 2021-1-X-2, and 2024-K097). All patients and legal representatives of minor patients provided written informed consent.

## Data Availability

The original data contributions to this article will be made available by the authors, without any reservation.
