# Supplementary material for: Intratonsillar Immunotherapy: A Convenient and Effective Alternative to Subcutaneous Immunotherapy for Allergic Rhinitis
Source: Research (Wash D C). 2025 Jan 16;8:0573. doi: 10.34133/research.0573 (PMC11735709; doi:10.34133/research.0573)

Table S1 Overall efficacy comparison and covariate adjustment

Characteristic		Post-treatment vs. baseline		SCIT vs. ITIT					
		SCIT P value	ITIT P value	Score			Difference from baseline		
				Unadjusted model P value	Adjusted model[1] P value	Covariate[2] impact on model P value	Unadjusted model P value	Adjusted model[1] P value	Covariate[2] impact on model P value
**VAS**	**1 month**	<0.0001	<0.0001	0.0883	0.1297	0.6131	0.0064	0.0163	0.8120
	**2 months**	<0.0001	<0.0001	0.1016	0.2163	0.4538	0.0248	0.0386	0.7884
	**3 months**	<0.0001	<0.0001	0.2451	0.2592	0.8872	0.0750	0.0719	0.6735
	**6 months**	<0.0001	<0.0001	0.1771	0.1471	0.5232	0.0489	0.1083	0.6861
	**1 year**	<0.0001	<0.0001	0.2958	0.2675	0.5413	0.3843	0.4340	0.9287
**SS**	**1 month**	0.0002	<0.0001	0.0839	0.0749	0.5827	0.0057	0.0149	0.8344
	**2 months**	<0.0001	<0.0001	0.0414	0.0399	0.4086	0.0417	0.0062	0.8260
	**3 months**	<0.0001	<0.0001	0.1808	0.1741	0.9300	0.0942	0.0785	0.7739
	**6 months**	0.0003	<0.0001	0.1424	0.1660	0.4515	0.0451	0.1069	0.6186
	**1 year**	<0.0001	<0.0001	0.1882	0.2249	0.5431	0.3741	0.4845	0.9683
**MS[3]**	**1 month**	0.3119	0.0275	0.0742	0.0733	0.7489	0.7642	0.7095	0.3093
	**2 months**	0.0644	0.0017	0.0436	0.0583	0.7716	1.0000	0.5627	0.1820
	**3 months**	0.0252	0.0016	0.7411	0.6246	0.5836	0.8576	0.8756	0.3764
	**6 months**	0.2165	0.0136	0.4663	0.3932	0.5415	0.9807	0.7806	0.6855
	**1 year**	0.2165	0.0027	0.2135	0.1754	0.4988	0.2322	0.1773	0.4351
**CSMS**	**1 month**	0.0012	<0.0001	0.0177	0.0204	0.5586	0.0326	0.0433	0.3774
	**2 months**	<0.0001	<0.0001	0.0014	0.0349	0.7564	0.0489	0.1431	0.2911
	**3 months**	<0.0001	<0.0001	0.0019	0.0047	0.6979	0.0745	0.1593	0.4401
	**6 months**	0.0028	<0.0001	0.0098	0.5393	0.4421	0.0834	0.1288	0.9523
	**1 year**	0.0002	<0.0001	0.0758	0.1212	0.4501	0.6672	0.4287	0.6218

[1]The analysis of covariance (ANCOVA) includes adjustment for baseline dust mite sIgE
[2]Baseline dust mite-sIgE
[3]The natural logarithm was applied to normalize the data, followed by ANCOVA

Figure S1 Box plots illustrating changes in immune markers in peripheral blood of SCIT patients over time

The left y-axis is presented on a log10 scale, while the right y-axis indicates patient groups. Arrows indicate significant decrement (downward) or increment (upward) of immunological parameters after treatment. P values < .01, and .05 are indicated with ** and *, respectively. EOS, eosinophils granulocyte; BAS, basophilic granulocyte; IFN, interferon; TNF, tumor necrosis factor; IL, interleukin; Ig, immunoglobulin; sIgE, specific IgE; SCIT, Subcutaneous Immunotherapy. Dust mite-sIgE= Der p-sIgE+ Der f-sIgE. Units: EOS, BAS: 10*9/L; EOS%, BAS%: %; cytokines: pg/mL; IgG, IgA: g/L; tIgE, Der p-sIgE, Der f-sIgE, Dust mite-sIgE: kU/L; Der p-sIgE/tIgE, Der f-sIgE/tIgE, Dust mite-sIgE/tIgE: unitless.

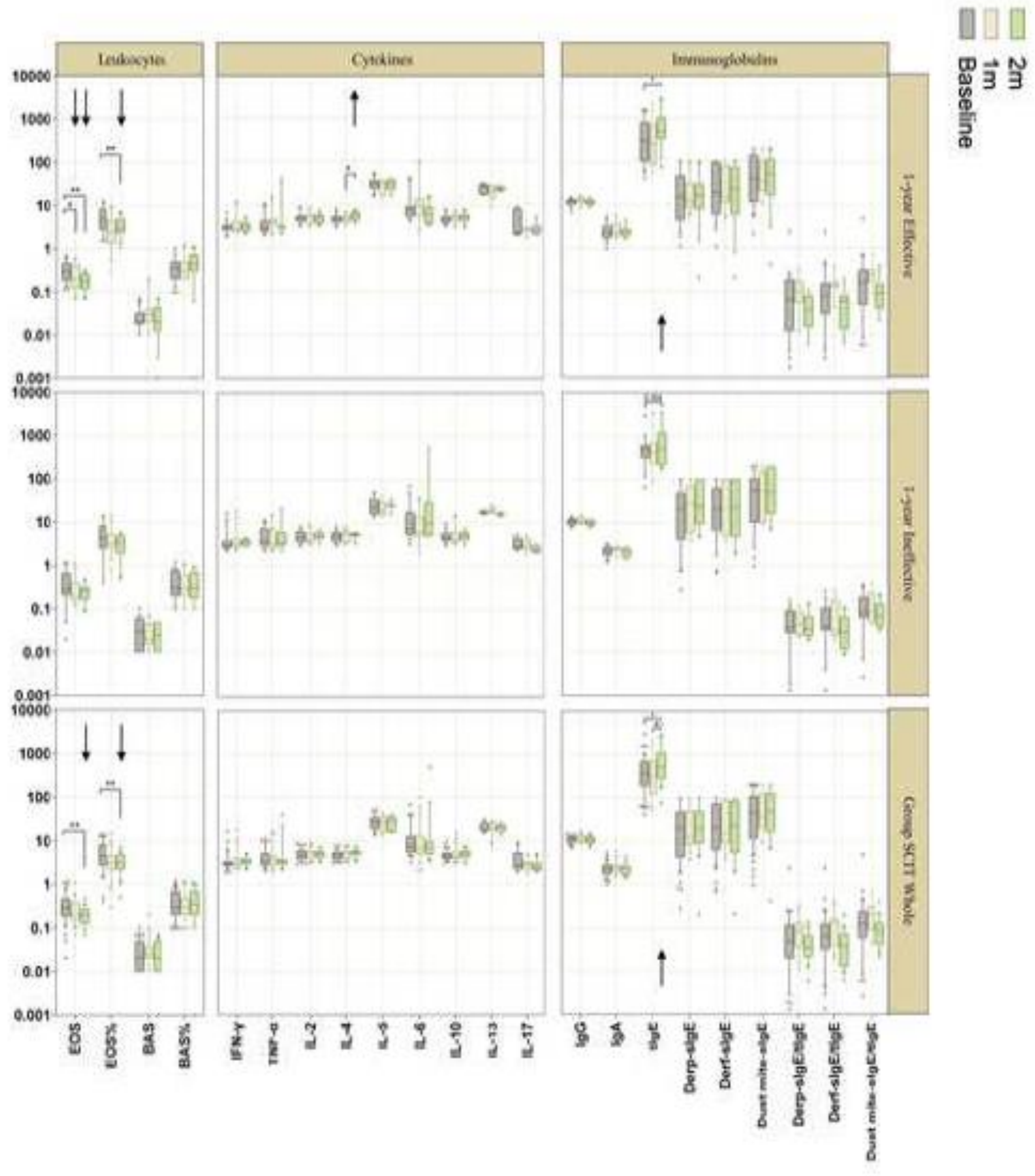

Supplement: Supplementary 1 — Fig. S1 Table S1 [file research.0573.f1.pdf]
